# Fano‐Like Acoustic Resonance for Subwavelength Directional Sensing: 0–360 Degree Measurement

**DOI:** 10.1002/advs.201903101

**Published:** 2020-01-27

**Authors:** Taehwa Lee, Tsuyoshi Nomura, Xiaoshi Su, Hideo Iizuka

**Affiliations:** ^1^ Toyota Research Institute of North America Toyota Motor North America Ann Arbor MI 48105 USA

**Keywords:** acoustic Fano resonances, coupled resonances, directional sensors

## Abstract

Directional sound sensing plays a critical role in many applications involving the localization of a sound source. However, the sensing range limit and fabrication difficulties of current acoustic sensing technologies pose challenges in realizing compact subwavelength direction sensors. Here, a subwavelength directional sensor is demonstrated, which can detect the angle of an incident wave in a full angle range (0°∼360°). The directional sensing is realized with acoustic coupling of Helmholtz resonators each supporting a monopolar resonance, which are monitored by conventional microphones. When these resonators scatter sound into free‐space acoustic modes, the scattered waves from each resonator interfere, resulting in a Fano‐like resonance where the spectral responses of the constituent resonators are drastically different from each other. This work provides a critical understanding of resonant coupling as well as a viable solution for directional sensing.

## Graphical abstract

Fano‐like resonances supported by deep‐subwavelength coupled resonators enable directional sensing. Owing to the coupled resonance, drastically different characteristics depending on incident angles are exhibited by the constituent resonators, which are used to identify the angle of incident acoustic waves in a full‐angle range.

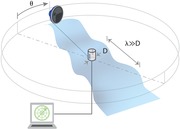

## 1 Introduction

Living creatures have evolved to perceive the world through their senses for their survival. Likewise, future society based on the convergence of the physical and the digital world will be fully indebted to sophisticated sensors to seemingly connect the two worlds. Among external stimuli, sound sensing is of great importance and is complementary to light‐based sensing owing to its characteristics such as omnidirectionality and relatively large wavelengths.^1^, ^2^ Particularly, directional sound sensing is of a critical component, enabling a myriad of applications including communication, navigation, and medical imaging. Due to the omnidirectional feature of sound detectors, directional sensing typically relies on two or more spaced‐apart detectors that can sense the difference in wave arrival time (or phase difference), making it difficult to use a compact subwavelength system and also requiring complicated signal processing associated beam forming.

Small creatures such as flies and lizards are known to possess the capability of accurately sensing the angle of an incident sound wave whose wavelength is much larger than their physical dimensions. Such an exotic characteristic in these small creatures has inspired miniature directional sensors both in optics^3^ and acoustics.^4^, ^5^, ^6^, ^7^, ^8^, ^9^, ^10^, ^11^ The mechanisms behind the subwavelength directional sensing are explained with coupled resonances that amplify acoustic energy stored in each resonance. For example, miniature MEMS directional sound sensors mimic the *Ormia ochracea* fly's hearing system where two eardrums of the fly are mechanically coupled, supporting dual vibration modes sensitive to the incident angle.^4^, ^5^, ^6^, ^7^ Moreover, for small animals (lizards), subwavelength sensing employs dipolar resonances through the two internally coupled eardrums (membranes) that permit instantaneous pressure difference across the membrane.^8^, ^9^, ^10^, ^11^ Despite their promising performance, the bioinspired directional sensors based on such an internal coupling or structurally coupled resonators pose challenges associated with dedicated sensing components and a limited sensing range (i.e., from 0° only up to 180°).

Pioneering metamaterial‐based devices exhibit superior capability of acoustic wave control^12^, ^13^, ^14^, ^15^, ^16^, ^17^, ^18^ and wave sensing.^1^, ^2^, ^19^, ^20^, ^21^, ^22^ Particularly, directional sound reception in a narrow angle range (rejecting noise from the other angles) is enabled by topological insulator with valley polarized edge states^19^ and phononic crystals.^20^, ^21^ In addition, acoustic Mie scatterers demonstrated azimuthally varying pressure field dependent on the incident angle, enabling to identify the incident angle by using a conventional microphone imbedded in the scatterer.^22^ In such a sensor, pressure gains relative to the incident pressure, characterized by a single microphone, is related to the incident angle in a range of 0°–90°. Besides the relatively narrow sensing angle range, the Mie scattering‐based sensing requires the knowledge of pressure at the device, thus limiting its implementation in scenarios such as sensing of amplitude‐varying acoustic waves (e.g., pulsed waves). In addition, the use of an additional reference microphone away from the sensing device compromises the compactness of the whole system.

Here, we demonstrate a subwavelength sensor that can detect the angle of an incident acoustic wave in a range of 0°–360°. The subwavelength directional sensing is enabled by radiatively coupled acoustic resonators. We find that the interference of scattered waves from the constituent resonators results in a Fano‐like resonance, where the resonators exhibit drastically different characteristics. By taking the acoustic power ratio of these resonators, the incident angle is identified without needing to know pressure at the device. First, we show a two‐resonator sensor for angles 0°–180° as a platform to analytically and experimentally investigate coupling of resonances. The analytical model based on coupled harmonic oscillators elucidates radiative coupling between the resonators. Lastly, we demonstrate a three‐resonator sensor having the extended sensing range of 0°–360°.

## 2 Results and Discussion

### 2.1 Coupled Resonance for Directional Sensing

**Figure 1:**
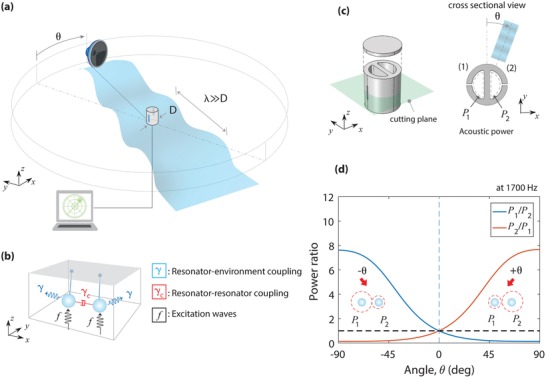
Acoustic subwavelength sensor for directional sensing. a) Illustration of sensing the angle of an incident acoustic wave using a cylindrical sensor composed of two acoustic resonators. Two microphones monitor the responses of the resonators for directional sensing. b) Illustration of harmonic oscillator model for direction sensor: the two harmonic oscillators coupled via radiative damping. c) Sound direction sensor consisting of a cylinder having two Helmholtz resonators. A difference in acoustic powers inside the cavities (*P*_1_ and *P*_2_) is related to the incident angle. d) Simulated angle‐dependent ratio of the acoustic powers characterized inside the lossless cavities at a specific frequency (1700 Hz) for the device (23 mm diameter, 25 mm height, 1 mm slit width, 10 mm slit height, 3 mm slit depth, and 3 mm wall thickness).

We investigate incident‐angle sensing illustrated in **Figure** **1**a, in which an acoustic sensor is composed of a subwavelength rigid cylinder decorated with two deep‐subwavelength resonators. By characterizing these resonators in response to an incident acoustic wave, the direction of the incident acoustic wave is identified in an angle‐sensing range of 0°−180° or −90°−90° (i.e., half angles). In such a subwavelength regime, the difference in arrival time of a sound between these two resonators is negligible, and coupling between the resonators plays a critical role in directional sensing. Our objective is to understand the coupling phenomena associated with the directional sensing. Beyond the half‐angle sensing range, we further conceive a sensor capable of sensing the full angle of an incident wave (i.e., 0°−360°).

To understand the role of the coupled resonances, we take a look at the behavior of the coupled resonators subjected to an incident external sound wave characterized by *f*, as illustrated in Figure 1b. Both resonators are simultaneously excited by an incident acoustic wave, yet the excitation at each resonator has a difference in phase angles characterized by 2*πd*sin(θ)/λ (*d*: distance, θ: incident angle). For a subwavelength scale (i.e., small *d*/λ), the excitation phase difference between the resonators is relatively small. Each subwavelength resonator couples to free‐space environment (characterized by γ), scattering incident waves through scattering channels. Besides, the scattered waves from each resonator interact with the other resonator, that is, radiatively coupling (denoted to γ_c_). Importantly, the radiative coupling amplifies the excitation difference, leading to a drastically different response of each resonator for directional sensing. The interference between the scattered fields from the constituent resonators contrives a Fano‐like resonant system having two resonators simultaneously excited, contrasting to typical Fano resonances where only one resonator is driven by an external force.^23^, ^24^, ^25^, ^26^, ^27^

### 2.2 Directional Subwavelength Sensor Design

We design a cylindrical sensor consisting of two‐sided Helmholtz resonators (HRs), each having a slit and a cavity, as shown in Figure 1c. Here, the two HRs spaced apart by a subwavelength distance are strongly coupled at resonance. These coupled resonators are similar to coupled membrane resonators in small animals' hearing systems, in which the membrane resonators yield dipolar resonances that are internally coupled.^8^, ^9^, ^10^, ^11^ In our case, HRs induce monopolar resonances and are only externally coupled, amplifying the acoustic power at resonance. The behaviors of the resonators can be readily characterized by a conventional microphone inserted into each cavity. Numerical simulations of the sensor were carried out using a commercial finite‐element‐method solver, COMSOL (see the Experimental Section). The acoustic powers within the cavities are characterized by *P*_1_ and *P*_2_, which are averaged over an area (highlighted by dashed lines in Figure 1c) by neglecting pressure variations inside the cavity. Such a negligible pressure variation is justified when the wavelength of the sound is much longer than the dimensions of the resonator.

The incident angle of acoustic waves is characterized by a difference in acoustic powers between the cavities 1 and 2. Figure 1d shows the numerically calculated acoustic power ratios (*P_ij_* = *P_i_* /*P_j_*) as a function of the incident angle (θ) in the two identical lossless resonators at *f* = 1700 Hz. In this sensing frequency, the device (23 mm diameter) is much smaller than the wavelength (λ = 201 mm). The *C*_2_ rotational symmetry of the sensor permits half angle sensing (−90°−90°), also exhibiting *P*_1_/*P*_2_ symmetrical to *P*_2_/*P*_1_ with respect to θ = 0°. For oblique incidence, the power ratios deviate from unity and gradually increases with |θ| despite the small excitation phase difference between the subwavelength resonators. Notably, the resonator close to the incident angle (i.e., resonator 2 for the positive angles θ > 0° and resonator 1 for θ < 0°) has significant power enhancement, exhibiting the maximum power ratio of almost 8 at θ = ± 90°.

### 2.3 Theoretical Treatment

The response of the two‐resonator sensor can be analyzed by modeling each HR as a harmonic oscillator consisting of a mass (*m*), damper (δ), and spring (*k*). For these coupled harmonic oscillators, the equation of motion is given by^28^, ^29^, ^30^

(1) $$\frac{{\text{d}}^{2}}{\text{d}t^{2}}\left|\begin{array}{ll} m & 0\\ 0 & m \end{array}\right|\left[\begin{array}{l} x_{1}\\ x_{2} \end{array}\right]+\frac{\text{d}}{\text{d}t}\left|\begin{array}{ll} \gamma +\delta & \gamma_{c}\\ \gamma_{c} & \gamma +\delta \end{array}\right|\left[\begin{array}{l} x_{1}\\ x_{2} \end{array}\right]+\left|\begin{array}{ll} k & 0\\ 0 & k \end{array}\right|\left[\begin{array}{l} x_{1}\\ x_{2} \end{array}\right]=\left[\begin{array}{l} f_{1}\\ f_{2} \end{array}\right]$$

where *x*_1_ and *x*_2_ represent the amplitudes of oscillators 1 and 2, respectively, and *f*_1_ and *f*_2_ are the external forces with the driving frequency ω. Here, the mass (*m*) of HR corresponds to a lump of air at the slit and the vicinity of the slit, which is given by *m* = *ρS*(*l* + *l*_c_) with ρ being the mass density, *S* the cross sectional area of the slit, *l* the slit length, and *l*_c_ the correction length for the air mass at the vicinity of the slit. In addition, the spring (*k*) corresponding to the springness of air inside the cavity is expressed by *k* = *ρcS* ^2^/*V* with *c* the speed of sound and *V* the cavity volume. Importantly, the diagonal elements of the damping matrix (i.e., δ and γ) indicates damping responsible for the decay of the vibrations in the resonators; γ is the radiation leakage describing coupling between a resonator and environment, while δ is the nonradiative loss occurring around the slits of HRs. The off‐diagonal elements of the damping matrix (γ_c_) that represents radiative coupling between oscillators 1 and 2.

By solving Equation (1) with the definition of *X_ij_* = *x_i_* /*x_j_*, the vibration amplitude of oscillator 1 (2) is represented by

(2) $$x_{1\left(2\right)}\;\left(\omega \right)\;\;=\;\;\frac{f_{1\left(2\right)}\left(\omega \right)/m}{\left[\omega_{0}^{2}-\omega^{2}+\frac{1}{m}\text{Im}\left(\gamma +\gamma_{\text{c}}X_{21\left(12\right)}\right)\omega \right]-i\frac{1}{m}\left[\delta +\text{Re}\left(\gamma +\gamma_{\text{c}}X_{21\left(12\right)}\right)\right]\omega }$$

where ω is the radian frequency, and ω_0_ is the natural frequency $\left(=\sqrt{k\text{/}m}\;\right)$. From Equation (2), we find that both γ and γ_c_ play a critical role in determining the response of the resonator, constituting an effective damping (i.e., γ_eff,1(2)_ =  γ + γ_c_ *X*_21(12)_). Note that the coupling effect is characterized by a combination of γ_c_ and *X*_21(12)_, and the magnitude of *X*_21(12)_ determines the strength of the coupling. The imaginary part of γ_eff_ is responsible for resonance shift, while the real part determines the vibration amplitude. In our formalism, both γ and γ_c_ can be analytically calculated (see the Experimental Section), providing a critical understanding of coupling phenomena.

Under assumptions of adiabatic volume changes and negligible pressure variations inside the cavity, pressure inside the cavity 1 is expressed with *x*_1_ in Equation (2) by

(3) $$p_{1}\;\;=\;\;-\gamma_{\text{s}}\frac{S_{1}}{V_{1}}x_{1}$$

where γ_s_ is the ratio of specific heats (1.4 for air). Using the acoustic power inside the cavity $\left(P_{1}\;\;=\;\;\frac{|p_{1}{|}^{2}}{2\rho c}\right)\;$, the power ratio of the signals is represented by *P*_1_/ *P*_2_ = |*x*_1_|^2^/|*x*_2_|^2^.

**Figure 2:**
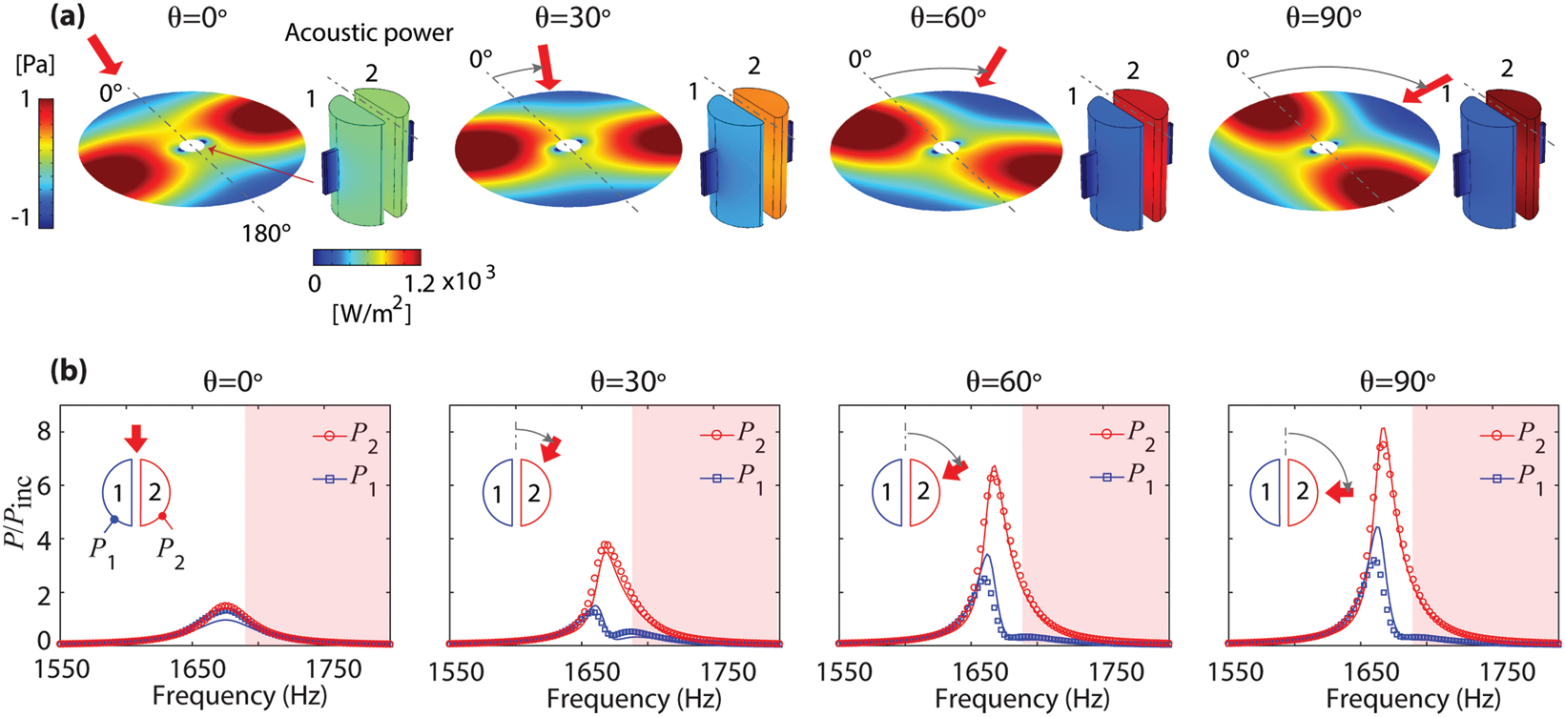
Angle‐dependent acoustic power ratio between the lossless cavities. a) Simulated acoustic fields outside the resonators and acoustic power inside the resonators for the incident angles of θ = 0°, 30°, 60°, and 90°. b) Analytically calculated and simulated acoustic power spectra of the two cavities for the incident angles. The solid lines indicate the analytical results using Equation (3), while the symbols indicate the simulation results. The parameters used in Equation (2) are given by *m*  =  6.96  × 10^‐6^ kg, *k*  =  9.04 N m^−1^, and δ  =  0 kg s^−1^. γ_c_ and γ are analytically calculated using Equation (6). *X*_21_ and *f_j_* are given by Equation (5) and Equation (7), respectively.

Our analytical model is validated with numerical analysis. **Figure** **2**a shows the numerical results of pressure fields around the sensor and acoustic power inside the cavities at a frequency of 1700 Hz for the incident angles of θ = 0°, 30°, 60°, and 90°. The device dimensions are given by 23 mm diameter, 25 mm height, 1 mm slit width, 10 mm slit height, 3 mm slit depth, and 3 mm wall thickness. For oblique angles (θ ≠ 0°), we observe the acoustic shadow effect that the acoustic power of the upstream resonator (i.e., cavity 2) greatly exceeds that of the downstream resonator (cavity 1). Figure 2b shows the acoustic power spectrum in each cavity, normalized to the incident acoustic power (*P*_inc_) for the lossless case (δ = 0). The analytical results (solid lines) obtained using Equation (3) show good agreement with the numerical results (symbols). In the analytical model, the effective mass and stiffness are used for HRs, which are given by *m* = 6.96 × 10^‐6^ kg, *k* = 9.04 N m^−1^ .γ_c_ and γ are analytically calculated using Equation (6) in Experimental Section, which are frequency‐dependent, complex numbers. Note that for oblique angles, the two resonators exhibit very different spectral line‐shapes; the cavity 2 (upstream) show a Lorentzian spectral shape, whereas the cavity 1 (downstream) has an asymmetric spectral line. Such a drastic spectral difference leads to the large power contrast (*P_i_*/*P_j_*), enabling high‐sensitive directional sensing. Here, the small discrepancy between the analytical and numerical results originates from inaccuracy of *l*_c_ used for *m*. Rigorous validation of coupling terms (γ and γ_c_) is discussed in the Supporting Information.

The directional sensing of the two‐resonator sensor is limited to a range of off‐resonance frequencies (red shade in Figure 2b) starting from 1680 Hz. At resonance, the acoustic power difference between the two resonators can be the largest. However, at frequencies close to the resonance frequency, one‐to‐one correspondence between θ and *P_i_*/*P_j_* is not guaranteed; *P_i_*/*P_j_* increases with |θ| up to a certain angle, and beyond it decreases because of angle‐dependent frequency bandwidths. Note that as increasing θ, the frequency bandwidth of the cavity 2 becomes narrower, and the frequency at its peak remains constant while the peak frequency of the cavity 1 is slightly blue‐shifted with increasing θ. In addition, acoustic waves do not cause significant structural vibration, because the eigenfrequencies of the device are located far away from the sensing frequencies (see the Supporting Information).

### 2.4 Fano‐Like Resonance for Angle Sensing

**Figure 3:**
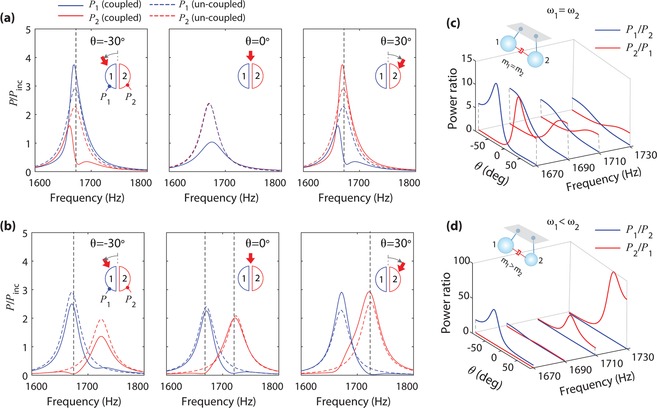
Directional Fano‐like resonance for angle sensing. a) Analytically calculated acoustic power of the identical resonators ( ω_1_ = ω_2_ ) characterized in each cavity for θ  =   − 30°, 0°, and 30°. The parameters used in the analytical model are given by   =  6.96  × 10^‐6^ kg, *k*  =  9.04 N m^−1^, and δ  =  0 kg s^−1^. The dashed line represents un‐coupled resonators (i.e., γ_c_ =  0). The acoustic power is normalized to the incident power. b) Acoustic power of the detuned resonators (ω_1_ ≠ ω_2_ through *m*_2_ =  0.93*m*_1_). c) Acoustic power ratio in the identical resonators ( ω_1_ = ω_2_ ). d) Acoustic power ratio in the detuned resonators (ω_1_ ≠ ω_2_).

The asymmetric spectral line‐shape in the downstream resonator, enabling a large *P_i_*/*P_j_*, results from a Fano‐like resonance due to interference between two scattering processes. **Figure** **3**a shows the analytically calculated acoustic power spectra of the resonators with and without coupling between the resonators (i.e., on‐ and off‐interference). Here, the response of the un‐coupled case (dashed line) is obtained by setting the off‐diagonal elements of the damping matrix to zero (i.e., γ_c_ = 0) in Equation (1). The comparison between the coupled and uncoupled cases clearly exhibits that the asymmetric spectral line‐shape is induced by the neighboring resonator; and its dip coincides with the peak of the other resonator (see the vertical dashed lines).

The Fano resonance states interference between a background and a resonant scattering process (i.e., interference between very different scattering processes). In our case, the interference happens between two resonators having the same resonance frequency (i.e., ω_1_ = ω_2_ ), which is similar to electromagnetically induced transparency (EIT) considered a special case of Fano resonance.^31^ Unlike EIT's unambiguous spectral lines due to γ_1_ ≠ γ_2_, our case exhibits the ambiguity in the interference effect due to γ_1_ = γ_2_ . Thus, to show more clear interference effects, we consider slightly detuned resonators (ω_2_ = ω_1_ + Δ), as shown in Figure 3b. Due to the detuning, the acoustic power spectrum of one resonator evidently shows the effect of the neighboring resonator.

In Figure 3a, the asymmetric spectral line‐shape and the dip formation are observed only for oblique incidence. We find that oblique incidence causes the same resonators to have slightly different resonance shifts, thus enabling the dip formation. From Equation (2), the resonance frequency (ω_res_) satisfies $\omega_{0}^{2}-\omega_{\text{res}}^{2}+\frac{1}{m}\text{Im}(\gamma +\gamma_{\text{c}}X_{21(12)})\;\omega_{\text{res}}=\;0$, which is characterized by ω_res,1(2)_ = ω_0_ + Δω_1(2)_. Here, the resonance shift (Δω) originates from the imaginary part of the effective damping term, i.e., Im(γ + γ_c_ *X*_21(12)_). It is obvious that detuning of the resonances (i.e., Δω_1_ ≠ Δω_2_) occurs when *X*_12_ ≠ *X*_21_ for oblique incidence.

The identical resonator shows symmetry between *P*_1_/*P*_2_ and *P*_2_/*P*_1_, as shown in Figure 3c for four different frequencies. However, the detuned resonators [Figure 3b] show an asymmetric characteristic in Figure 3d. Notably, the detuned resonators having dips of *P_i_* ≈ 0 leads to an extremely large *P_i_*/*P_j_*, enabling a high detection sensitivity, as the sensitivity is proportional to the slope of the *P_i_*/*P_j_* curve, i.e., d*P_ij_*/dθ. Particularly, an extremely large contrast of *P*_2_/*P*_1_ ≈ 100 is observed for θ = 40° at 1730 Hz, which is an order of magnitude higher than that of the identical resonators. Thus, the detuned resonators can be useful in some limited applications requiring high sensitivity in a narrower angle range.

**Figure 4:**
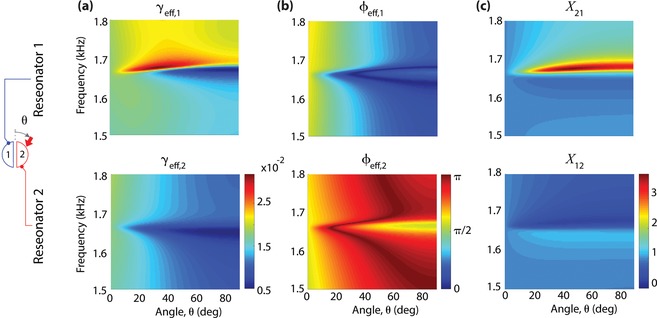
Angle‐dependent interference of the two resonators. a) Effective damping for the positive incident angles (θ > 0) for resonators 1 (top panel) and 2 (bottom panel). b) Difference of phase angles between radiation leakage and coupling. c) Ratio of oscillation amplitudes between two resonators.

The Fano‐like resonance of the coupled resonators is quantitatively described by an effective damping term, γ_eff,1(2)_ = Re(γ + γ_c_ *X*_21(12)_) in Equation (2). We observe a considerable difference of γ_eff_ between the resonators, as shown in **Figure** **4**a. Note that the upstream resonator (i.e., resonator 2 for positive angles) shows a very low damping γ_eff_ responsible for a high acoustic power, whereas the downstream resonator (resonator 1) has a relatively high γ_eff_ with a sharp transition corresponding to the dips of the spectral lines. Such a drastically different effective damping results from the interference between radiation leakage (γ) and radiative coupling (γ_c_ *X*_21(12)_): either constructive or destructive. As shown in Figure 4b, we find that the very low γ_eff_ of the resonator 2 is realized by the destructive interference, i.e., ϕ_eff,2_ = |Arg(γ) − Arg(γ_c_ *X*_12_)| ≈ π. In other words, the leakage damping (γ) of the resonator 2 is cancelled out by the coupling damping (γ_c_ *X*_12_), leading to the decrease in γ_eff_. The sharp transition of γ_eff,1_ across the resonance frequencies (Figure 4a) is responsible for the dip of the downstream resonator, which is induced by the change in the strength of coupling, i.e., *X*_21_, as shown in Figure 4c.

### 2.5 Experimental Demonstration of Directional Sensing

**Figure 5:**
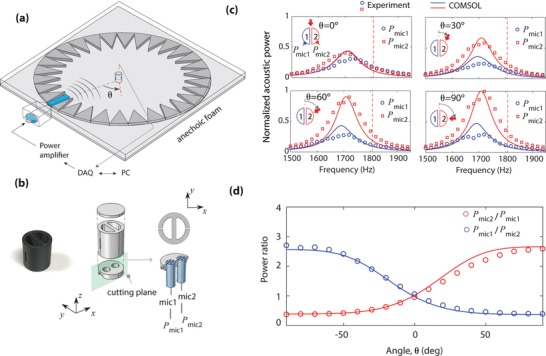
Measurement of the incident angle using the two‐resonator sensor. a) Illustration of the measurement setup consisting of the sensor placed at the center of a 2D waveguide with radial anechoic termination. b) Schematics of the two‐resonator sensor (perspective view and cross‐sectional view). c) Spectra of two microphone signals for the incident angles (θ  =  0°, 30°, 60°, and 90°), normalized to the maximum of *P*_mic,2_ at θ  =  90°. The symbols indicate the measurement data, while the solid lines indicate the simulation data. The insets show the incident angle (red arrow) with respect to the two resonators. The red vertical dashed lines indicate the frequency, where the acoustic power ratio is calculated. d) Angle‐dependent power ratio at *f*  =  1820 Hz.

To experimentally demonstrate sensing of the incident angles by considering realistic losses in the resonators (δ ≠ 0), the two‐resonator sensor (25 mm height) is placed in a 2D waveguide having the same height, as illustrated in **Figure** **5**a. For different incident angles, the sensor mounted on a motorized rotation stage is rotated, while the point sound source emitting cylindrical waves is fixed. Thus, the precision rotation stage controls the incident angle, and the measurement setup is shown in the Supporting Information. To measure the acoustic powers inside the cavities, two microphones are inserted through the holes on the bottom plate, as the photo of a 3D‐printed device and the insertion of the microphones are shown in Figure 5b. The dimensions of the device are found in the Experimental Section.

Figure 5c shows the acoustic power spectra measured with two microphones for θ = 0°, 30°, 60°, and 90°. Considering the thermoviscous losses in the resonators, the numerical results (solid lines) are in a fair agreement with the measurement results (symbols). Due to the intrinsic loss, the acoustic power spectra of the resonators exhibit a shifted resonance at *f* = 1700 Hz, compared to the lossless case (Figure 2b). Despite the intrinsic losses, the maximum power ratio reaches almost 3 for θ = ± 90° as shown in Figure 5d at 1820 Hz (see *P*_ij,max_ = 8 for the lossless case in Figure 1d). At this operating frequency, the sensor is much smaller than the wavelength (i.e., λ/8). Note that the sensing sensitivity proportional to the slope (i.e., d*X*_21(12)_/dθ) shows the highest for angles close to normal, and it decreases as |θ| approaches 90°. The sensing sensitivity over the entire angles is shown in the Supporting Information.

### 2.6 Full Angle Sensing by Three‐Resonator Sensor

**Figure 6:**
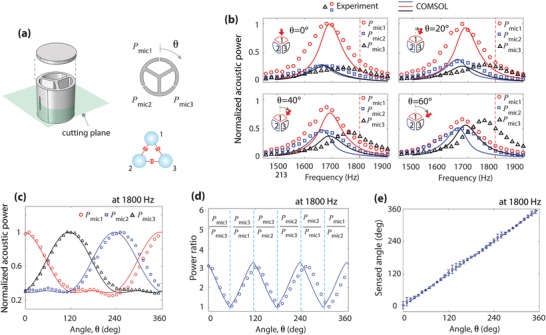
Three‐resonator sensor for full incident angles (0°–360°). a) Schematics of the three‐resonator sensor (perspective view and cross‐sectional view). b) Spectra of three microphone signals for the incident angles (θ  =  0°, 20°, 40°, and 60°), normalized to the maximum of *P*_mic,1_ at θ  =  0°. The symbols indicate the measurement data, while the solid lines indicate the simulation data. The insets show the incident angle (red arrow) with respect to the three resonators. c) Angle‐dependent acoustic power measured by the three microphones at *f*  =  1800 Hz. Each curve is normalized to its peak. The simulation results (solid lines) are plotted with the measurement results (symbols). d) Acoustic power ratios of the data shown in (c). Full angles are divided into the six divisions (i.e., every 60°). The power ratio in each division is calculated with the two microphones having higher signals, as labeled in the figure (e.g., *P*_mic,1_/*P*_mic,3_ for the first division). e) Detected angles with respect to the incident angles. The symbols represent the mean values for five measurements, while the error bars indicate the largest deviation from the mean value.

To overcome the sensing range limit imposed by the two‐resonator sensor, we conceive a sensor consisting of three identical resonators (26 mm diameter), as illustrated in **Figure** **6**a. The three‐resonator sensor having *C*_3_ rotational symmetry enables full angle sensing (i.e., 0°−360°). Compared to the two‐resonator sensor, coupling phenomena occurring in the three‐resonator sensor are more complicated, which are characterized by the effective damping modified for three resonators (e.g., γ_eff,1_ =  γ + γ_c_ *X*_21_ + γ_c_ *X*_31_ for the resonator 1). The acoustic power spectra measured by three microphones are plotted in Figure 6b for θ = 0°, 20°, 40°, and 60°. The acoustic powers from all three microphones are normalized to the peak value of *P*_mic1_ at θ = 0°. The simulation results (solid lines) capture the trend of the measurement results (symbols). The discrepancy between the measurement and simulation results is associated with fabrication accuracy, as discussed in the Supporting Information. Similar to the two‐resonator sensor, the three‐resonator sensor shows the acoustic shadowing effect, i.e., the acoustic power of the two resonators away from the sound source (i.e., resonators 2 and 3) is much smaller than that of the first resonator close to the source. Note that the resonators 2 and 3 in the acoustic shadow exhibit a slightly different characteristic, allowing to distinguish between − θ and + θ. With increasing θ, such a shadowing effect is diminished, and for an incident angle of θ = 60°, all three resonators show similar acoustic powers (Figure 6b).

Figure 6c shows the acoustic powers with respect to the incident angle θ at a frequency of 1800 Hz (higher than the resonance frequencies; see the red vertical dashed line in Figure 6b). The numerical results show good agreement with the measured acoustic powers normalized to each microphone's peak value. The good agreement proves that the proposed sensing method is robust despite the discrepancy observed in Figure 6b. Each microphone has the maximum acoustic power when an acoustic wave is directly incident on the corresponding resonator (i.e., mic 1 for θ = 0°, mic 2 for θ = 120°, and mic 3 for θ = 240°). For directional sensing, we take an acoustic power ratio between the first two highest values at a specific angle (e.g., for θ = 30°, *P*_13_ = *P*_mic1_ /*P*_mic3_), as plotted in Figure 6d. Note that the three‐resonator sensor demonstrates almost constant sensing sensitivity (constant slope) throughout all the angles. The sensing sensitivity over the entire angles is found in the Supporting Information. Figure 6e shows the directional sensing performance. The symbols indicate the measurement data averaged with the five measurements (error bars showing the largest error of all the measurements), while the black dashed line indicates the sensed angles equal to the incident angles. The large sensing errors (up to ± 7.9^°^) are observed for the angles around 0^°^, 120^°^, and 240^°^, because the acoustic signals from the downstream microphones are low, and thereby sensitive to the environment noise.

**Figure 7:**
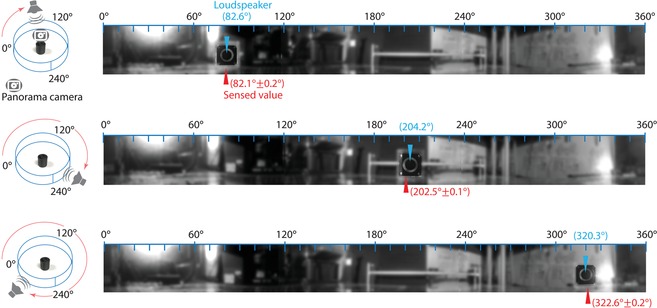
Sensing the incident angle of acoustic waves in free space. 360° panoramic photos of a sound source (i.e., loudspeaker) of different radial positions (θ  =  82.6°, 204.2°, and 320.3°; indicated as blue arrows). The incident angles detected by the three‐resonator sensor are displayed on the images. The detected values are represented as (mean ± standard deviation) for ten measurements. The corresponding schematics show perspective views of the sensor and loudspeaker for different incident angles. The panoramic camera is placed at the same position as the sensor. The background of the panorama images are intentionally blurred for clear identification of the loudspeaker (see the original images in the Supporting Information).

The measurements so far have been conducted using the sensors placed inside the 2D waveguide. When the sensor is used in open space, the characteristics of the sensors are changed, because the constituent resonators have different leakage (γ) and coupling terms (γ_c_) from those in the 2D waveguide. **Figure** **7** shows the directional sensing performance of the three‐resonator sensor located in open space (3D field). For three different positions of a loudspeaker, panorama photos are taken and overlaid with the measured angles (mean ± standard deviation) with respect to the actual locations of the speaker. The angular positions are determined from the images, and the background is intentionally blurred such that the loudspeaker is clearly identified (see the original images in the Supporting Information). The results for the chosen angles demonstrate a reasonable sensing accuracy, while the angles around 0^°^, 120^°^, and 240 have relatively high sensing errors. The sensing accuracy can be improved by choosing a sensing frequency closer to the resonance frequency or implementing a four‐resonator device, as discussed in the Supporting Information.

## 3 Conclusion

We have demonstrated subwavelength directional sensing based on the Fano‐like resonance. The proposed sensors consisting of coupled resonators decorating a rigid cylinder can sense the angle of an incident acoustic wave in a range of −90°−90° for the two‐resonator sensor or 0°−360° for the three‐resonator sensor. From an interference perspective, the Fano‐like resonance enabling directional sensing is explained with the analytical model based on the coupled harmonic oscillator model. The experimental demonstration proves the usefulness of the compact directional sensor. As our sensing method based on the acoustic power ratio does not require the knowledge of pressure at the device, it is applicable to incident acoustic waves of which amplitudes vary with time (e.g., pulsed waves). This work does not only provide a critical understanding of radiatively coupled resonance phenomena, but also offers a viable sensing solution using conventional microphones.

## 4 Experimental Section

##### Numerical Simulations

All simulations were conducted by using a commercial finite‐element‐method (FEM) solver (COMSOL Multiphysics 5.3). The intrinsic loss of a HR is considered by using the Thermoviscous Acoustic Interface in COMSOL Multiphysics.

##### Analytical Model

To derive Equation (2), the damping matrix of Equation (1) can be diagonalized as

(4) $$\frac{\text{d}}{\text{d}t}\left|\begin{array}{cc} \gamma +\delta & \gamma_{\text{c}}\\ \gamma_{\text{c}} & \gamma +\delta \end{array}\right|\;\left[\begin{array}{c} x_{1}\\ x_{2} \end{array}\right]\;\;=\;\;\frac{\text{d}}{\text{d}t}\;\left|\begin{array}{cc} \gamma +\delta +\gamma_{\text{c}}X_{21} & 0\\ 0 & \gamma +\delta +\gamma_{\text{c}}X_{12} \end{array}\right|\left[\begin{array}{c} x_{1}\\ x_{2} \end{array}\right]$$

By solving Equation (1) with the diagonalized damping matrix, *x*_1_(ω) and *x*_2_(ω) are determined. The ratio of $\frac{x_{2}(\omega )}{x_{1}(\omega )}$ is expressed by

(5) $$X_{21}\;\;=\;\;\frac{x_{2}\left(\omega \right)}{x_{1}\left(\omega \right)}\;\;=\;\;\left[\frac{\left(-\omega \text{/}\omega_{0}+\omega_{0}\text{/}\omega \right)\gamma_{0}+2i\left(\delta +\gamma -f_{12}\gamma_{\text{c}}\right)}{\left(-\omega \text{/}\omega_{0}+\omega_{0}\text{/}\omega \right)\gamma_{0}+2i\left(\delta +\gamma -f_{21}\gamma_{\text{c}}\right)}\right]\;f_{21}$$

with *f_ij_* being the ratio of the external forces ( *f_ij_* = *f_i_* /*f_j_*) and γ_0_ being the critical damping 2(km)^1/2^.

By integrating the scattered pressure field from each resonator *j* (*p*_res,*j*_) over an area of *S_i_* (resonator *i*), the coupling terms (γ and γ_c_) are analytically determined by

(6) $$\gamma_{ij}=\frac{\int_{S_{i}}p_{\text{res},j}\left(\vec{r}\right)\text{d}S}{v_{j}\;}\;=\frac{i\omega \rho w^{2}}{2\pi k_{\text{w}}R}\;\sum_{n\;=\;-\infty }^{\infty }\frac{H_{n}\left(k_{w}R\right)}{H_{n}^{}\prime \left(k_{\text{w}}R\right)}e^{in\left(\theta_{i}-\theta_{j}\right)}$$

where *v_j_* is the velocity of the resonator *j*, *k*_w_ is the wavenumber, ρ is the mass density, *w* is the slit width, *R* is the radius of the cylinder, *H_n_* is the *n*th order Hankel function of the first kind. When *i* = *j*, Equation (6) leads to the leakage rate (i.e., γ_*ij*_ = γ). When *i* ≠ *j*, γ_*ij*_ = γ_*c*_. The excitation force acting on resonator *j* is expressed by^32^, ^33^

(7) $$f_{j}=\int_{S_{j}}\left[p_{\text{int}}\left(r\right)+p_{\text{dir}}\left(r\right)\right]dS$$

where *p*_int_ is the incident pressure field, and *p*_dir_ is the scattered pressure field from the nonresonant rigid cylinder. *p*_dir_ is given by

(8) $$p_{\text{dir}}\;\left(r,\;\theta \right)\;\;=\;\;-p_{0}\sum_{n\;=\;-\infty }^{\infty }i^{n}\frac{J_{n}^{}\prime \left(k_{w}R\right)}{H_{n}^{}\prime \left(k_{w}R\right)}H_{n}\left(k_{\text{w}}r\right)e^{in\theta }$$

where (*r*, θ) are the polar coordinate, *p*_0_ is the incident pressure amplitude, and *J_n_* is the *n*th order Bessel function of the first kind.

##### Measurement

The sensors were printed by a 3D printer (Model: Markforged Mark Two, Markforged Inc. MA, USA) with 400 µm nozzle diameter and 100 µm layer height. The printing material is made of a chopped carbon fiber filled nylon composite with 1.4 GPa tensile modulus and 1.18 g cm^−3^ density. The parts were additively manufactured with a 100% fill factor without continuous fiber reinforcement. The dimensions of the two‐resonator sensor are given by *H* = 25 mm, *D* = 23 mm (diameter), *h* = 10 mm (slit height in the z direction), *w* = 1 mm (slit width), *l* = 3 mm (slit depth), and *t* = 3 mm (thickness of the walls), while the dimensions of the three‐resonator sensor are given by *H* = 25 mm, *D* = 26 mm (diameter), *h* = 10 mm (slit height in the *z* direction), *w* = 1 mm (slit width), *l* = 2 mm (slit depth), and *t* = 2 mm (thickness of the walls).

The measurement setup consists of a 2D waveguide made of top/bottom acrylic sheets (1/2″ thick, 48″ × 48″), and a full‐range speaker (2½″, model SB65WVAC25‐4, SB Acoustics, https://www.sbacoustics.com/) connected to a square waveguide (20 × 20 × 150 mm^3^). The test devices were mounted to a motorized rotation stage (PRMTZ8, Thorlabs, New Jersey, USA). Information related to the rotation control is found in the Supporting Information. Each device contains two pressure‐field microphones (1/4″ prepolarized, sensitivity 1 mV Pa^−1^, model 378C10, PCB Piezotronics, NY, USA). The audio power amplifier (model APA150, Daytonaudio), data acquisition device (24‐bit, 102.4 kS s^−1^, model NI USB‐4431, National Instruments) are used. The signals acquired from microphones were Fourier‐transformed to check the incident frequency and to reject the effect of other frequencies.

## Conflict of Interest

The authors declare no conflict of interest.

## Supporting information

Supporting Information

## References

[1] Y. Chen , H. Liu , M. Reilly , H. Bae , M. Yu , Nat. Commun. 2014, 5, 5247. 25316410 10.1038/ncomms6247

[2] Y. Xie , T. H. Tsai , A. Konneker , B. Popa , D. J. Brady , S. A. Cummer , Proc. Natl. Acad. Sci. USA 2015, 112, 10595. 26261314 10.1073/pnas.1502276112 PMC4553806

[3] S. Yi , M. Zhou , Z. Yu , P. Fan , N. Behdad , D. Lin , K. X. Wang , S. Fan , M. Brongersma , Nat. Nanotechnol. 2018, 13, 1143. 30374161 10.1038/s41565-018-0278-9

[4] M. Touse , J. Sinibaldi , K. Simsek , J. Catterlin , S. Harrison , G. Karunasiri , Appl. Phys. Lett. 2010, 96, 173701.

[5] M. L. Kuntzman , N. A. Hall , Appl. Phys. Lett. 2014, 105, 033701.

[6] D. Wilmott , F. Alves , G. Karunasiri , Sci. Rep. 2016, 6, 29957. 27440657 10.1038/srep29957 PMC4954978

[7] H. Liu , L. Currano , D. Gee , T. Helms , M. Yu , Sci. Rep. 2013, 3, 2489. 23966060 10.1038/srep02489 PMC3749551

[8] J. Christensen‐Dalsgaard , G. A. Manley , J. Exp. Biol. 2005, 208, 1209. 15767319 10.1242/jeb.01511

[9] J. Christensen‐Dalsgaard , Y. Tang , C. E. Carr , J. Neurophysiol. 2011, 105, 1992. 21325679 10.1152/jn.00004.2011 PMC3094191

[10] C. Vossen , J. Christensen‐Dalsgaard , J. L. van Hemmen , J. Acoust. Soc. Am. 2010, 128, 909. 20707461 10.1121/1.3455853

[11] A. P. Vedurmudi , J. Goulet , J. Christensen‐Dalsgaard , B. A. Young , R. Williams , J. L. van Hemmen , Phys. Rev. Lett. 2016, 116, 028101. 26824568 10.1103/PhysRevLett.116.028101

[12] S. A. Cummer , J. Christensen , A. Alù , Nat. Rev. Mater. 2016, 1, 16001.

[13] B. Assouar , B. Liang , Y. Wu , Y. Li , J. Cheng , Y. Jing , Nat. Rev. Mater. 2018, 3, 460.

[14] J. Li , C. T. Chan , Phys. Rev. E 2004, 70, 055602(R). 10.1103/PhysRevE.70.055602 15600684

[15] H. Chen , C. T. Chan , Appl. Phys. Lett. 2007, 91, 183518.

[16] Z. Yang , J. Mei , M. Yang , N. H. Chan , P. Sheng , Phys. Rev. Lett. 2008, 101, 204301. 19113343 10.1103/PhysRevLett.101.204301

[17] S. Koo , C. Cho , J. Jeong , N. Park , Nat. Commun. 2016, 7, 13012. 27687689 10.1038/ncomms13012 PMC5056457

[18] S. H. Lee , C. M. Park , Y. M. Seo , Z. G. Wang , C. K. Kim , Phys. Rev. Lett. 2010, 104, 054301. 20366767 10.1103/PhysRevLett.104.054301

[19] Z. Zhang , Y. Tian , Y. Wang , S. Gao , Y. Cheng , X. Liu , J. Christensen , Adv. Mater. 2018, 30, 1803229. 10.1002/adma.201803229 30059167

[20] T. Jiang , Q. He , Z. Peng , Appl. Phys. Lett. 2018, 112, 261902.

[21] C. Ma , S. Gao , Y. Cheng , X. Liu , Appl. Phys. Lett. 2019, 115, 053501.

[22] X. Zhu , B. Liang , W. Kan , Y. Peng , J. Cheng , Phys. Rev. Appl. 2016, 5, 054015.

[23] B. Luk'yanchuk , N. I. Zheludev , S. A. Maier , N. J. Halas , P. Nordlander , H. Giessen , C. T. Chong , Nat. Mater. 2010, 9, 707. 20733610 10.1038/nmat2810

[24] M. F. Limonov , M. V. Rybin , A. N. Poddubny , Y. S. Kivshar , Nat. Photonics 2017, 11, 543.

[25] Y. Sun , J. Xia , H. Sun , S. Yuan , Y. Ge , X. Liu , Adv. Sci. 2019, 6, 1901307. 10.1002/advs.201901307 PMC6794620 31637167

[26] F. Zangeneh‐Nejad , R. Fleury , Phys. Rev. Lett. 2019, 122, 014301. 31012649 10.1103/PhysRevLett.122.014301

[27] W. Wang , Y. Jin , W. Wang , B. Bonello , B. Djafari‐Rouhani , R. Fleury , Phys. Rev. B 2020, 101, 024101.

[28] T. Lee , T. Nomura , E. M. Dede , H. Iizuka , Phys. Rev. Appl. 2019, 11, 024022.

[29] T. Lee , H. Iizuka , New J. Phys. 2019, 21, 043030.

[30] S. S. Rao , Mechanical Vibrations, 5th ed., Prentice Hall, Englewood Cliffs, NJ, USA 2011.

[31] Y. Yang , I. I. Kravchenko , D. P. Briggs , J. Valentine , Nat. Commun. 2014, 5, 5753. 25511508 10.1038/ncomms6753

[32] T. Lee , H. Iizuka , Phys. Rev. B 2019, 99, 064305.

[33] T. Lee , T. Nomura , P. Schmalenberg , E. M. Dede , H. Iizuka , Phys. Rev. Appl. 2019, 12, 054059.

