# Supplementary material for: Fano‐Like Acoustic Resonance for Subwavelength Directional Sensing: 0–360 Degree Measurement
Source: Adv Sci (Weinh). 2020 Jan 27;7(6):1903101. doi: 10.1002/advs.201903101 (PMC7080540; doi:10.1002/advs.201903101)
Supplement: Supplementary file 1 — Supporting Information [file ADVS-7-1903101-s001.pdf]

Copyright WILEY-VCH Verlag GmbH & Co. KGaA, 69469 Weinheim, Germany, 2020.

Supporting Information

for *Adv. Sci.*, DOI: 10.1002/advs.201903101

Fano-Like Acoustic Resonance for Subwavelength Directional Sensing: 0–360 Degree Measurement

Taehwa Lee, Tsuyoshi Nomura, Xiaoshi Su, and Hideo Iizuka*

Copyright WILEY-VCH Verlag GmbH & Co. KGaA, 69469 Weinheim, Germany, 2018.

Supporting Information

Fano-like acoustic resonance for subwavelength directional sensing: 0-360 degrees measurement

Taehwa Lee, Tsuyoshi Nomura, Xiaoshi Su, and Hideo Iizuka*

Note List:

Note 1. Validation of coupling damping γ_c……………………………………………………..……. page. 2

Note 2. Effect of fabrication tolerance on the device performance……………………………. page. 3

Note 3. Four-resonator device for directional sensing……………………………………..……. page. 4

Note 4. Effect of structure-acoustic interaction…………………………………………………. page. 5

Note 5. Sensitivity of the devices………………………………………………………………. page. 6

Note 6. Experimental setup……………………………………………………………………. page. 6

Note 7. Original panorama images……………………………………………………………. page. 7

Note 1. Validation of coupling damping $\boldsymbol{\gamma_c}$.

The coupled resonators are modeled with the coupling force terms ($F_{i\to j}$, i.e., force exerting on resonator i, generated from resonator j):

$$m\frac{d^2x_1}{dt^2}+(\gamma+\delta)\frac{dx_1}{dt}+F_{2\to 1}+kx_1=f_1, \qquad (S1a)$$

$$m\frac{d^2x_2}{dt^2}+(\gamma+\delta)\frac{dx_2}{dt}+F_{1\to 2}+kx_2=f_2. \qquad (S1b)$$

To determine the coupling force $F_{2\to 1}$, consider pressure acting on resonator 1, generated from resonator 2 (i.e., $p_{2\to 1}$), which is expressed by [S1]

$$p_{2\to 1}=-\frac{i\omega\rho}{4\pi}\int_{S2}\frac{dx_2}{dt}GdS, \qquad (S2)$$

where G is the Green's function. By using $F_{2\to 1}=\int_{S_1}p_{2\to 1}dS$ with S_1 (area of resonator 1), the coupling force is given by

$$F_{2\to 1}=-\frac{i\omega\rho}{4\pi}\int_{S_1}\int_{S2}\frac{dx_2}{dt}GdS\,dS=-\frac{i\omega\rho}{4\pi}\int_{S_1}\int_{S2}GdS\,dS\frac{dx_2}{dt}=\gamma_c\frac{dx_2}{dt}. \qquad (S3)$$

Here, the coupling term is defined by $\gamma_c=-\frac{i\omega\rho R}{4\pi}\int_{S_1}\int_{S2}GdS\,dS$. G is expressed by $-\frac{2}{k_wR}\sum_{n=-\infty}^{\infty}\frac{H_n(k_wR)}{H_n'(k_wR)}e^{in(\theta_1-\theta_2)}$. For a resonator with a narrow slit, a pressure variation on the slits is neglected, i.e., $\int_{S_1}\int_{S2}GdS\,dS$=$G\int_{S_1}\int_{S2}dS\,dS=w^2$ for unit depth (2D wave propagation). Thus, coupling damping is represented by

$$\gamma_c=\frac{i\omega\rho w^2}{2\pi k_wR}\sum_{n=-\infty}^{\infty}\frac{H_n(k_wR)}{H_n'(k_wR)}e^{in(\theta_i-\theta_j)}. \qquad (S4)$$

Here, γ is calculated by using Equation (S4) for $\theta_i-\theta_j=0$.

To validate γ_c, COMSOL simulation using a lossless spring-mass system is compared with the analytical result, as shown in **Figure S1**a. In the COMSOL simulation (acoustic and structure modules), mass (m), stiffness (k), and intrinsic loss (δ) are explicitly defined without using fitting parameters, as illustrated in Figure S1b. The parameters used for COMSOL simulation and analytical model are given by m =1.2×10^6 kg, k =419.26 N/m, $\delta=0$ kg/s.

[S1] C. Lagarrigue, J. P. Groby, V. Tournat, O. Dazel, and O. Umnova, Absorption of sound by porous layers with embedded periodic arrays of resonant inclusions, J. Acoust. Soc. Am. 134 (6), 4670 (2013).

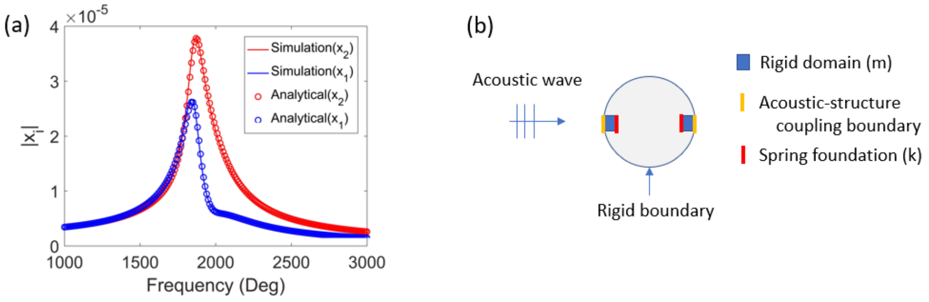

Figure S1. a) Comparison of the resonators' responses ($|x_i|$) between COMSOL simulation and analytical results. b) COMSOL model using a lossless mass-spring system. The parameters are given by m =1.2×10^6 kg, k =419.26 N/m, $\delta = 0$ kg/s.

Note 2. Effect of fabrication tolerance on the device performance.

Figure S2 shows the effect of a small dimension error (ε) for the slits induces a resonance shift. For ε =50 μm, a resonance shift is ~30 Hz, and for ε =100 μm, a resonance shift is ~60 Hz. By assuming ε =100 μm for the resonator 3, the experimental and simulation results show better agreement, as shown in **Figure S3**.

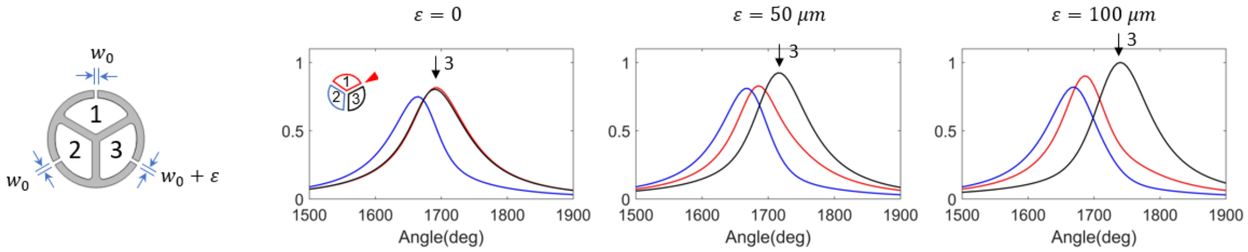

Figure S2. Effect of slit dimension errors (ε) for the resonator 3.

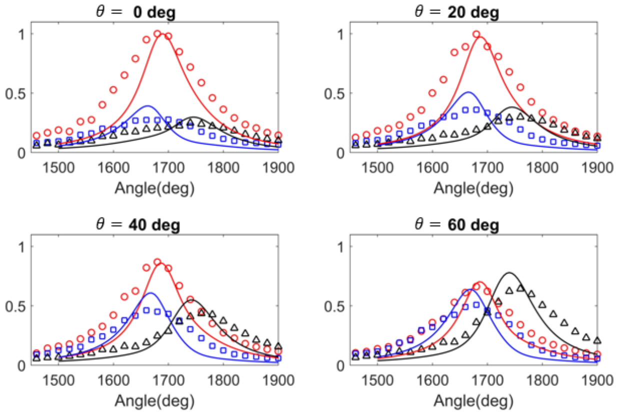

Figure S3. Re-plot of Figure 6b with an assumption of ε =100 μm.

Note 3. Four-resonator device for directional sensing.

Based on coupled resonators, a four-resonator device can be constructed, as illustrated in **Figure S4a**. For fixed slit dimensions, the size of the device is increased to D =30 mm. COMSOL simulation results are plotted in Figure S4b. In this four-resonator device, the acoustic power ratio is calculated from either the first two large signals or the first & third signals within each angle division (dashed vertical lines for every 60 degrees), as shown in Figure S4c,d. Figure S4d show relatively high $P_{mic,j}/P_{mic,i}$, but one-to-one correspondence is not ensured.

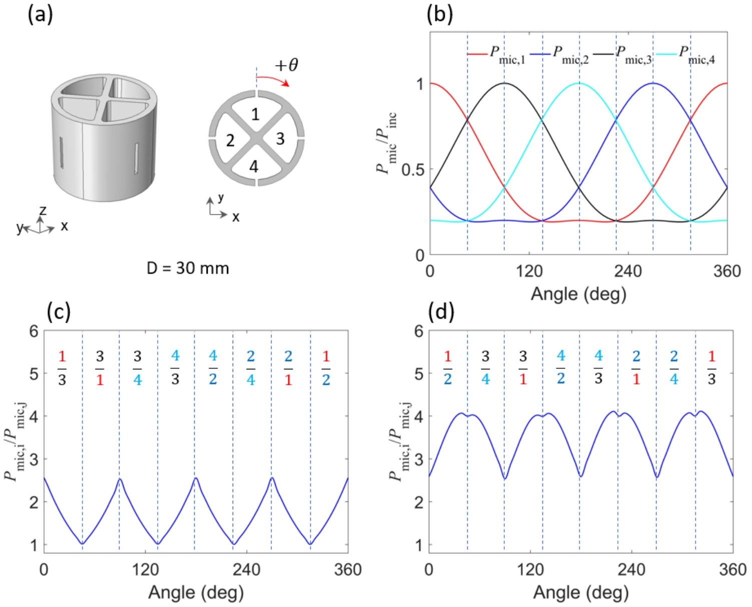

Figure S4. a) Four-resonator device (D = 30 mm). b) Simulated angle dependence at 1750 Hz. c) Power ratio between the first two. The numbers indicate the microphone signals used for nominators and denominators. d) Power ratio between the first and third.

Note 4. Effect of structure-acoustic interaction.

Figure S5 shows simulation results with and without considering acoustic-structure interaction. The rigidity of the plastic material (E=1.4GPa, ρ =1180 kg/m^3, ν =0.42) is sufficient. Mode analysis confirms that the eigenfrequencies of the two-resonator and three-resonator devices are far away from the sensing frequencies, as shown in **Figure S6**.

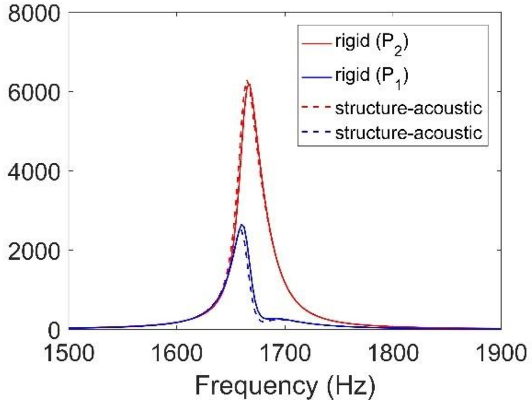

Figure S5. Effect of structure-acoustic interaction.

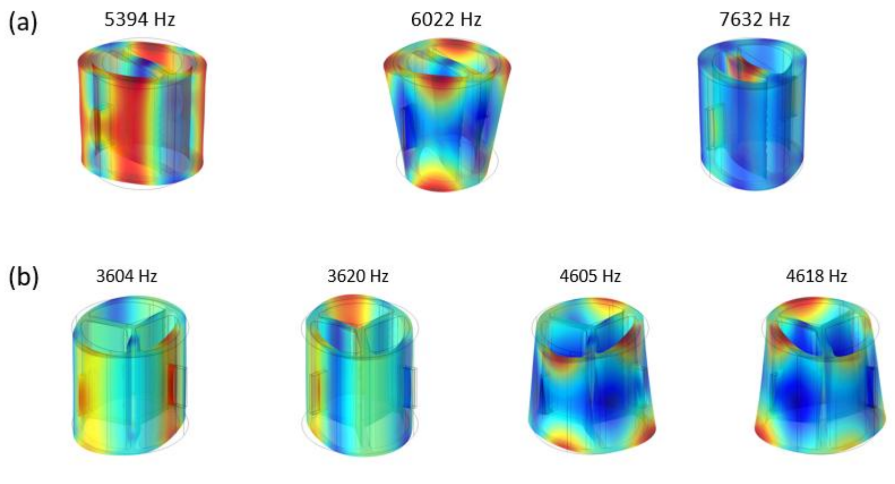

Figure S6. Mode analysis. a) Eigenfrequencies of the two-resonator device. b) Eigenfrequencies of the three-resonator device.

Note 5. Sensitivity of the devices.

The sensing sensitivity is defined by $|\frac{P_{ij}}{d\theta}|$, i.e., the slopes of Figure 5d and Figure 6d. The sensitivity of the two-resonator and three-resonator devices is plotted in **Figure S7**. For the two-resonator device, the sensitivity decreases with $|\theta|$. For the three-resonator device, the sensitivity is higher over the entire angles except angles of 60, 120, 180, 240, 300, and 360 degrees. The abrupt drop in sensitivity is not a critical issue in typical applications. If $\left|\frac{P_{ij}}{d\theta}\right| > 0.01$ is enough for rejecting environment noise, the sensible angle ($\delta\theta$) is 1.2 degree.

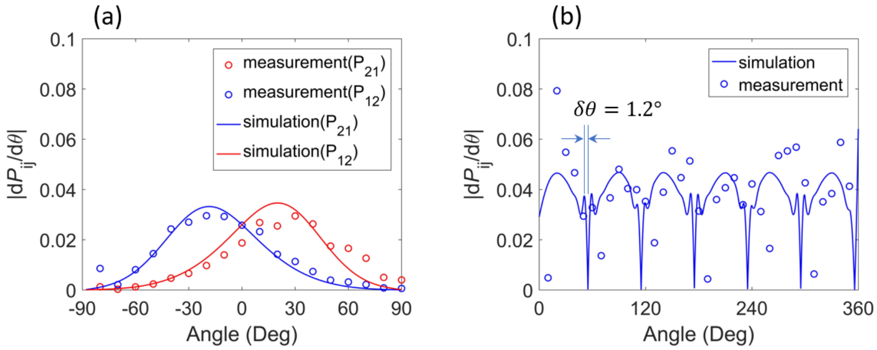

Figure S7. Sensitivity of the devices. a) Two-resonator device. b) Three-resonator device.

Note 6. Experimental setup.

While the sound source (i.e., loudspeaker) is fixed, the sensing device is precisely rotated by using a motorized rotation stage. Thus, the rotation angle defines the incident angle. In **Figure R11**, both microphones and device mounted on a motorized precision rotation stage are accurately rotated.

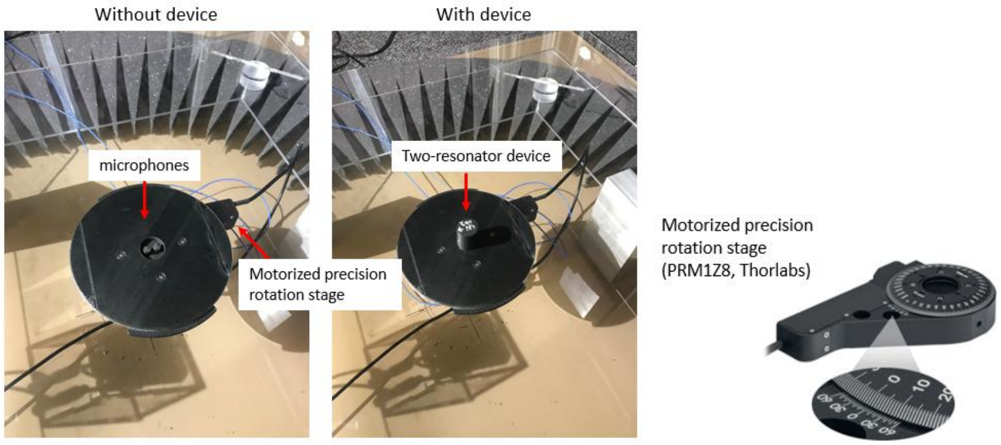

Figure S8. Measurement setup for rotational control of the device.

Note 7. Original panorama images.

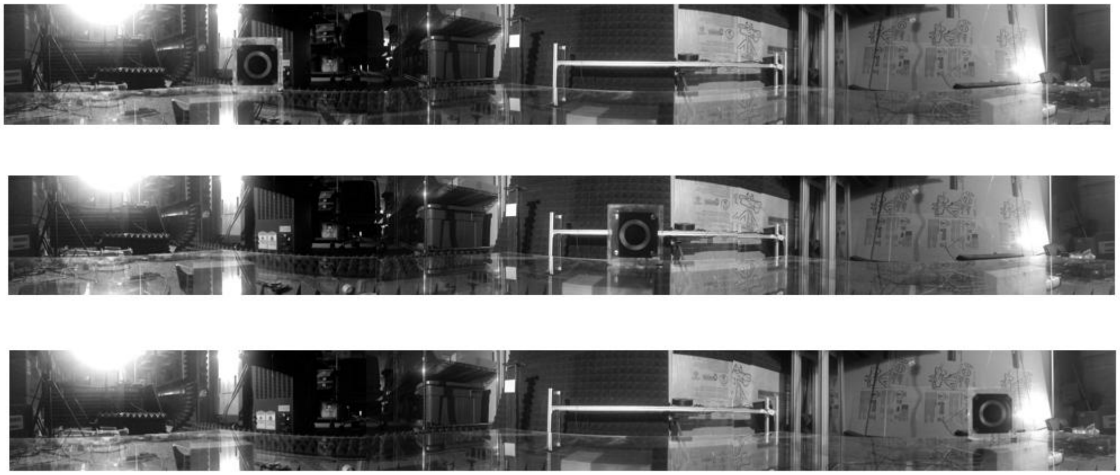

Figure S9. Original images without blurring the background.
